# Assessment of normal pulmonary development using functional magnetic resonance imaging techniques

**DOI:** 10.1016/j.ajogmf.2023.100935

**Published:** 2023-06

**Authors:** Carla L. Avena-Zampieri, Jana Hutter, Maria Deprez, Kelly Payette, Megan Hall, Alena Uus, Surabhi Nanda, Anna Milan, Paul T. Seed, Mary Rutherford, Anne Greenough, Lisa Story

**Affiliations:** 1 Department of Women and Children's Health, King's College London, London, United Kingdom (XX Avena-Zampieri, XX Hall, XX Seed, XX Greenough, and XX Story); 2 Centre for the Developing Brain, School of Biomedical Engineering & Imaging Sciences, King's College London, London, United Kingdom (Ms Avena-Zampieri, Dr Hutter, Mr Deprez, Ms Payette, Dr Hall, Ms Uus, Prof Rutherford, and Dr Story); 3 Fetal Medicine Unit, Guy's and St Thomas’ NHS Foundation Trust, London, United Kingdom (Dr Hall, Dr Nanda, and Dr Story); 4 Neonatal Unit, Guy's and St Thomas’ NHS Foundation Trust, London, United Kingdom (Dr Milan); 5 Neonatal Unit, King's College Hospital, London, United Kingdom (Prof Greenough); 6 National Institute for Health and Care Research Biomedical Research Centre based at Guy's & St Thomas NHS Foundation Trusts and King's College London, London, United Kingdom (Prof Greenough); 7 Department of Biomedical Engineering, School of Biomedical Engineering & Imaging Sciences, King's College London, London, United Kingdom (Ms Deprez, Ms Payette, and Ms Uus)

**Keywords:** fetal magnetic resonance imaging, lung development, prenatal prediction, T2*

## Abstract

**BACKGROUND:**

The mainstay of assessment of the fetal lungs in clinical practice is via evaluation of pulmonary size, primarily using 2D ultrasound and more recently with anatomical magnetic resonance imaging. The emergence of advanced magnetic resonance techniques such as T2* relaxometry in combination with the latest motion correction post-processing tools now facilitates assessment of the metabolic activity or perfusion of fetal pulmonary tissue in vivo.

**OBJECTIVE:**

This study aimed to characterize normal pulmonary development using T2* relaxometry, accounting for fetal motion across gestation.

**METHODS:**

Datasets from women with uncomplicated pregnancies that delivered at term, were analyzed. All subjects had undergone T2-weighted imaging and T2* relaxometry on a Phillips 3T magnetic resonance imaging system antenatally. T2* relaxometry of the fetal thorax was performed using a gradient echo single-shot echo planar imaging sequence. Following correction for fetal motion using slice-to-volume reconstruction, T2* maps were generated using in-house pipelines. Lungs were manually segmented and mean T2* values calculated for the right and left lungs individually, and for both lungs combined. Lung volumes were generated from the segmented images, and the right and left lungs, as well as both lungs combined were assessed.

**RESULTS:**

Eighty-seven datasets were suitable for analysis. The mean gestation at scan was 29.9±4.3 weeks (range: 20.6–38.3) and mean gestation at delivery was 40±1.2 weeks (range: 37.1–42.4). Mean T2* values of the lungs increased over gestation for right and left lungs individually and for both lungs assessed together (*P*=.003; *P*=.04; *P*=.003, respectively). Right, left, and total lung volumes were also strongly correlated with increasing gestational age (*P*<.001 in all cases).

**CONCLUSION:**

This large study assessed developing lungs using T2* imaging across a wide gestational age range. Mean T2* values increased with gestational age, which may reflect increasing perfusion and metabolic requirements and alterations in tissue composition as gestation advances. In the future, evaluation of findings in fetuses with conditions known to be associated with pulmonary morbidity may lead to enhanced prognostication antenatally, consequently improving counseling and perinatal care planning.

**AJOG MFM at a Glance**

**Why was this study conducted?**

This study was conducted to assess normal fetal pulmonary development across gestation using an advanced functional magnetic resonance imaging technique, T2*, potentially reflective of oxygenation and alterations in pulmonary tissue composition across gestational age.

**Key findings**

We demonstrated that mean T2* values increased between 20 and 38 weeks gestation.

**What does this add to what is known?**

This study provides original motion-corrected morphologic and functional assessment of the antenatal fetal lung during normal development. This will form the basis for reference ranges to assess fetuses who may have compromised pulmonary growth and function.

## Introduction

Normal lung development encompasses 5 principal stages and disruption at any of these time points can result in mortality or significant long-term pulmonary morbidity. Lung growth commences during the embryonic stage (3–7 weeks of development). This initial stage is characterized by vascularization and formation of smooth muscle within the airways, tracheal cartilage, and pleura. The second, pseudoglandular stage encompasses the 7 to 16-week period. During this time, formation of the conducting airways to the level of the terminal bronchioles is complete and 75% of bronchial branching has occurred.^1^ Vascularization increases during the cannicular stage (weeks 16–28) and by the end of this period, gaseous exchange becomes feasible. Furthermore, during this time, the first zones with an alveolar-capillary barrier start to develop. The period between 28 and 36 weeks, the saccular stage, is a transitional period where the terminal sacs form and biochemical lung maturation occurs. Type II pneumocytes now secrete surfactant, which reduces pulmonary surface tension. Mature alveoli do not form until after birth, the last stage of lung growth (alveolar) comprises development of the final alveolar sacs and is accompanied by a gradual increase in lung volume.

Current assessment of the fetal lungs in clinical practice is limited to the evaluation of size, most commonly using 2-dimensional ultrasound measures but also via 3-dimensional volumes derived either from ultrasound or magnetic resonance imaging (MRI).^2^, ^3^, ^4^ Although these assessment tools are useful and have been validated for prognostication of congenital diaphragmatic hernia, their clinical utility is limited given their paucity in demonstrating accurate prognostic value in other pulmonary disorders such as pulmonary hypoplasia associated with second trimester preterm prelabor rupture of the membranes.^5^

Fetal MRI is a rapidly evolving field and recent advances include bespoke techniques for both acquisition and analysis. These include the application of functional acquisition techniques, such as T2* relaxometry, as well as the emergence of sophisticated motion correction pipelines, including deformable slice-to-volume reconstruction (DSVR).^6^^,^^7^ T2* relaxation refers to the decay of transverse magnetization seen with gradient-echo (GRE) sequences.^8^ T2* relaxometry utilizes the fact that oxygenated and deoxygenated hemoglobin have different paramagnetic properties; and therefore the technique provides an indirect assessment of tissue perfusion. Mean T2* quantitative values can be acquired for a specific region of tissue and a map can be generated illustrating regional variations of oxygenation. This technique has already been utilized during pregnancy to investigate placental pathology associated with conditions such as preeclampsia and chronic hypertension.^9^^,^^10^ Normal T2* values have also been evaluated in healthy pregnancies in the second and third trimesters in fetal organs such as the brain^11^ and liver.^12^ However, only 1 small study to date, comprising of only 9 participants, has evaluated the fetal lungs using T2* relaxometry.^13^

This project aims to comprehensively assess fetal mean pulmonary T2* values across gestation in a large cohort of uncomplicated pregnancies to assess normal pulmonary development.

## Materials and Methods

A retrospective study was performed by selecting a control cohort from 3 studies ([REC: 16/LO/1573, IRAS 201609], [19/LO/0736, IRAS 253500], and [REC: 21/SS/0082, IRAS 293516]), undertaken at St Thomas’ Hospital over a 4-year period from 2018 to 2022. Inclusion criteria included the following: women with singleton, uncomplicated pregnancies with no preexisting medical conditions who subsequently delivered their babies after 37 weeks’ gestation with no neonatal complications. Exclusion criteria for MRI included women who had claustrophobia or a recently sited metallic implant. Cases with a birthweight centile <3rd or >97th centile (calculated using the INTERGROWTH centiles),^14^ because these may have had underlying pathology, were excluded. Cases were additionally excluded where MRI data was found to be corrupted or subject to excessive motion or where outcome data was incomplete.

All women underwent imaging on a clinical 3T Philips MRI scanner using a 32-channel cardiac coil in a supine position.^15^ Heart rate and intermittent blood pressure readings were performed during the scan and frequent verbal interaction was maintained. Demographic, delivery, and postnatal data, including maternal age, ethnicity, body mass index, gestational age at delivery, birthweight and neonatal outcome of all pregnancies were collected from clinical maternity and infant records with participant consent.

Anatomic imaging of the fetal thorax was performed using a T2-weighted single-shot turbo spin echo sequence acquired in 3 orthogonal planes focused on the fetal body and an additional whole uterus coronal plane.^16^ After acquisition of a B0 map, manual image-based shimming was performed using an in-house tool.^17^ A multiecho gradient echo single-shot echo planar sequence was then performed with the following parameters: 3mm^3^ resolution, 5 echo times (13.8 ms/70.4 ms/127.0 ms/183.6 ms/ 240.2 ms), repetition time (TR)=3 seconds, parallel imaging SENSE factor=3, flip angle=90^o^. The field of view was set to 360 mm × (320–400) mm × (60–120) mm using a coronal imaging plane aligned to the scanner coordinates, to encompass the whole thorax and minimize artifacts from parallel imaging reconstruction techniques. The imaging protocol, including additional techniques such as diffusion MRI, dynamic imaging, and perfusion imaging, was completed within 1 hour in all cases and women were offered a break midway through.

**Figure 1:**
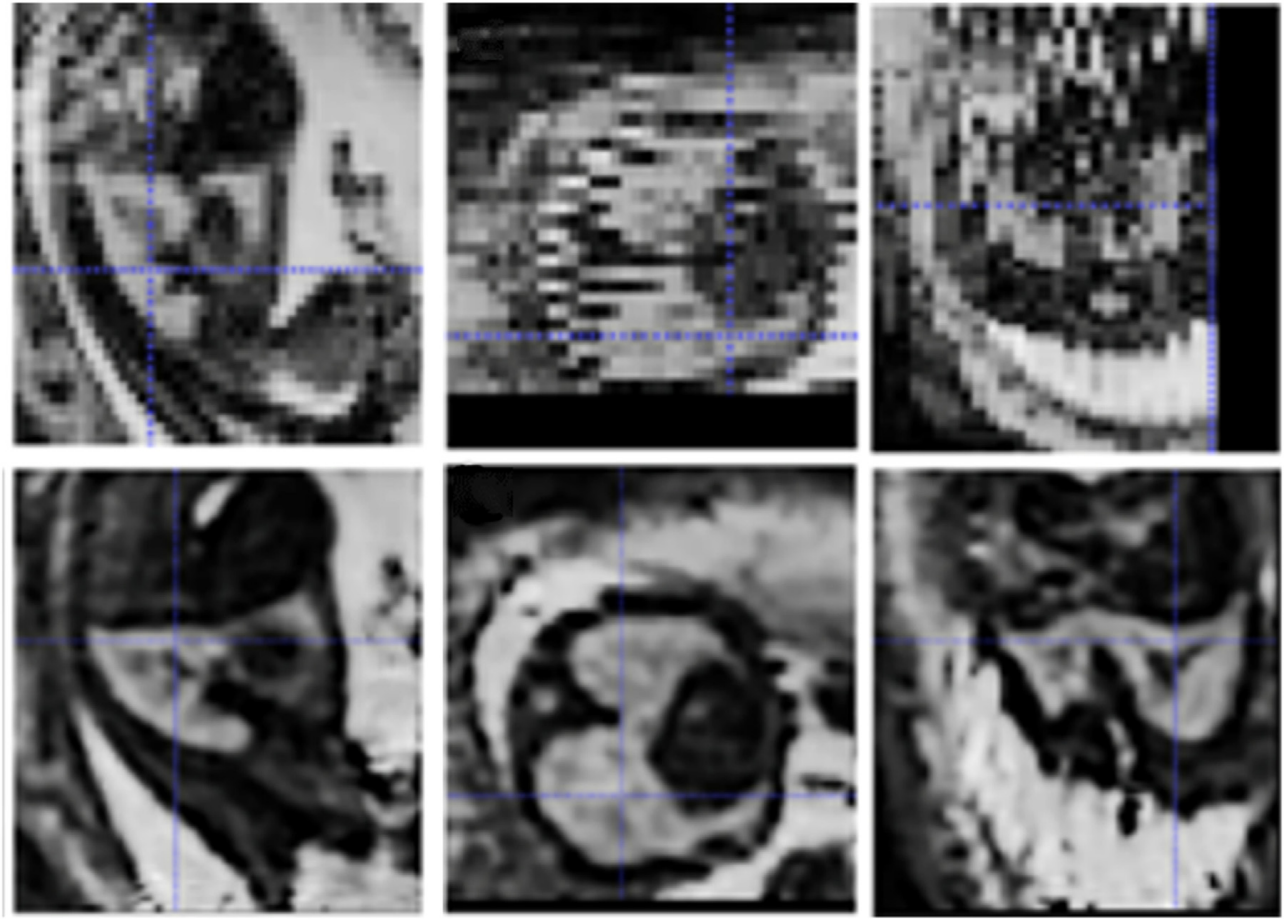
MRI images of the fetal thorax acquired on a 3 Tesla system *Row A* illustrates the native slice-plane and *row B* illustrates the corresponding view after deformable slice-to-volume reconstruction. The *first column* indicates the sagittal plane, the *second column* the axial plane and the *third column* the coronal plane.

As previously described, an in-house Python script was used to generate T2* maps using mono-exponential decay fitting.^16^ Another in-house pipeline, DSVR,^18^ has been previously used to generate reconstructed abdominal and thoracic images to account for unpredictable fetal motion. This technique was previously validated in fetal MRI for volumetric analysis from multiple stacks of slices,^6^ and was applied to the datasets. It facilitates correction of both in-plane and out-of-plane local deformations of fetal organs caused by bending and stretching motions and hence renders segmentations more accurate (Figure 1, A).

Avena-Zampieri. Assessment of normal pulmonary development. Am J Obstet Gynecol MFM 2023.

**Figure 2:**
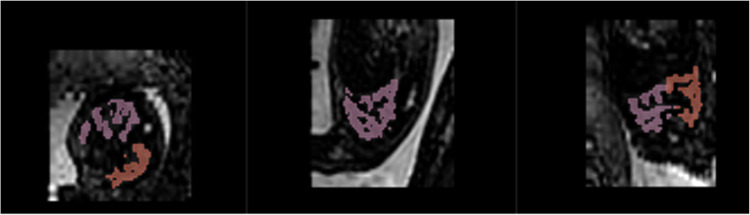
3D segmentation of fetal lung tissue in axial, sagittal and coronal planes The *pink color* illustrates right lung tissue and *red color* illustrates left lung tissue (segmented conservatively to avoid the vasculature in *black*).

Raw stacks used for reconstruction with satisfactory quality (where motion artifact was evident or the imaging of the thoracic region was incomplete were discarded) and where data for both lungs was available, were inspected and selected. The motion patterns obtained from the DSVR were applied to the spatially matched T2* maps. The resulting reconstructed images (an example of which is seen in Figure 1, B) were also inspected for adequate quality. The adequate 3D reconstruction quality implies minimal impact of fetal motion (if some remains), clear definition and visibility of both lungs without motion, sufficient for detailed delineation of lung tissue. Lung tissue segmentation was performed using the computer software 3D Slicer^19^ on the 3D reconstructed images. Manual segmentation of the lungs was performed by an experienced observer carefully avoiding any nonpulmonary tissue (Figure 2) (intensity range was adjusted to adapt the visualization and make segmentation easier by increasing contrast between pulmonary tissue and other structures, for example vascular structures and amniotic fluid).

Avena-Zampieri. Assessment of normal pulmonary development. Am J Obstet Gynecol MFM 2023.

Mean T2* values, lung volumes, and lacunarity scores (reflective of changes in granularity and heterogeneity of tissue^20^) were obtained and corresponding histograms were generated using a purpose-build Python script.^16^^,^^21^ In addition, T2* maps were assessed visually to provide a perceptible representation of heterogeneity of tissue oxygenation within the lungs. Cases were selected to give a visual representation of the changes over gestational age by selecting 1 per week to display the main lung stages of pulmonary development.

Applying the same segmentation methodology, we additionally obtained mean pulmonary T2* values from the raw images from 10 cases as well as the reconstructed images to assess the influence that motion correction had on the absolute T2* values.

Intraobserver and interobserver variability assessment of pulmonary T2* values, volumes, and their 95% confident intervals, were calculated using an absolute-agreement 2-way mixed-effects model in SPSS statistical package version 28.0.1 (SPSS IBM). Further statistical analysis was performed using Scipy in Python 3. Linear regression analysis was performed for lung volumes and T2* values in both lungs, as well as right and left lungs separately, to assess the relationship between gestational age and pulmonary parameters. In addition, parameters for the right and left lungs were compared. Ten of the women had a maximum of 2 scans during their pregnancies. We therefore accounted for serial measures by adjusting the standard errors and for clustering. The results remained significant, all demonstrating that both mean and standard deviation (SD) increase with gestation. In addition, we conducted an analysis, which led to a method for converting lung T2* to a *z*-score ([observed fetal T2*−mean]/SD for a given result), which can be converted to centiles using normal distribution. Centiles were derived from converting lung T2* values to *z*-scores and yielded the following for the mean: 1.488884*(gestational age of scan in weeks)+21.997645 and SD: 1.488884*(gestational age of scan in weeks)−18.451005.

## Results

**Figure 3:**
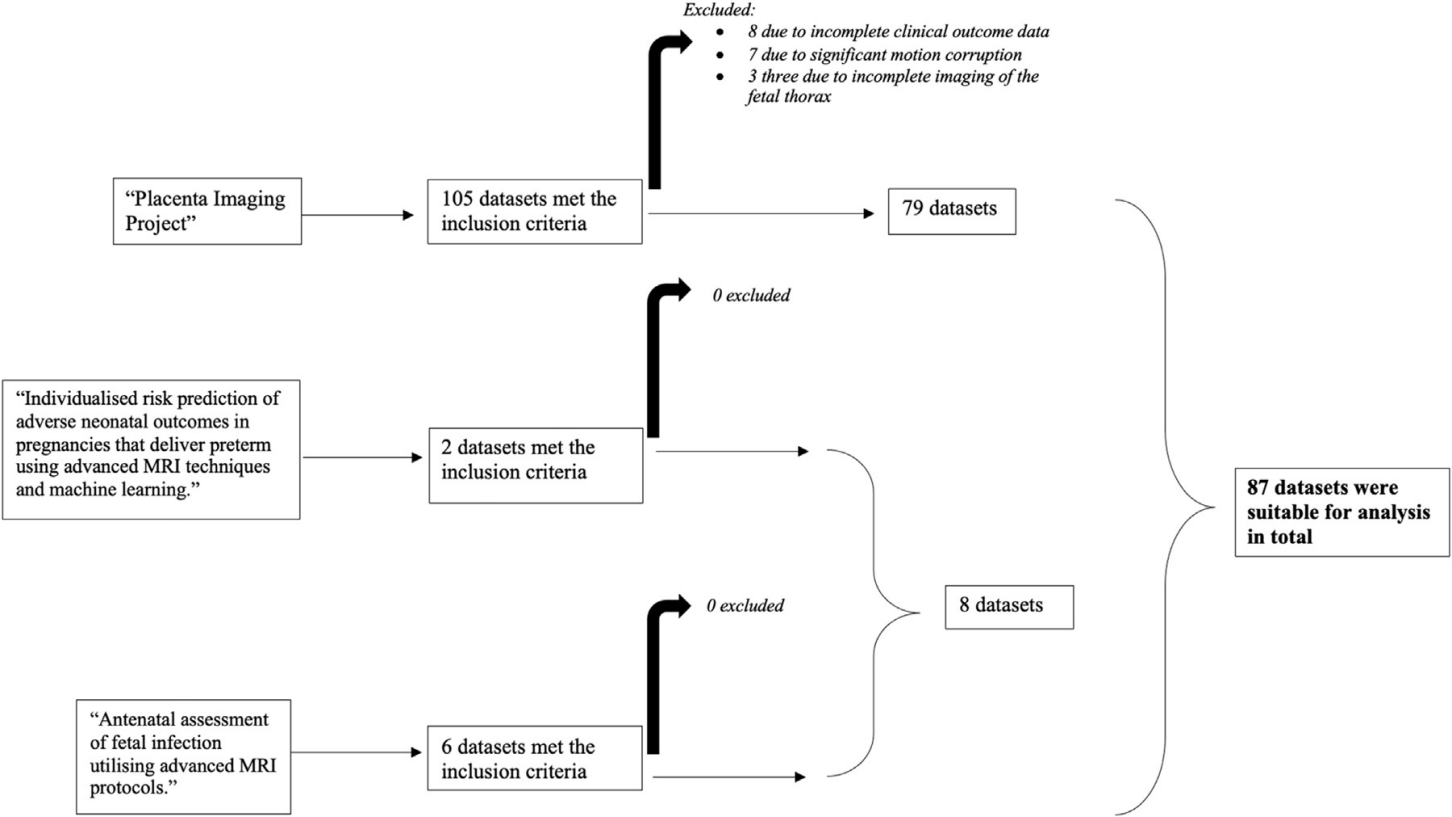
Flowchart of the data selection process

Eighty-seven datasets were suitable for analysis. Eighteen were excluded as follows: 3 owing to incomplete clinical outcome data; 7 owing to significant motion corruption, for which the motion correction pipelines could not compensate; and 3 owing to incomplete imaging of the fetal thorax (because the fetal thorax was not the primary region of interest of 2 of the studies from which data were included) (Figure 3). Maternal demographics and neonatal outcomes of the cohort are presented in Table. All neonates had an uncomplicated course post-delivery and did not require any form of respiratory support.

Avena-Zampieri. Assessment of normal pulmonary development. Am J Obstet Gynecol MFM 2023.

**Table:** Clinical characteristics study cohort

Maternal age (y)	
Mean (SD)	34 (3.7)
Range	25–45
Ethnicity n (%)	
White	79 (91%)
Mixed	1 (1%)
Asian	5 (6%)
Black	2 (2%)
BMI (kg/m^2^)	
Mean (SD)	22.4 (2.6)
Range	18.4–32.5
GA at scan (wk)	
Mean SD	29.9 (4.3)
Range	20.6–38.3
GA at delivery (wk)	
Mean SD	40 (1.2)
Range	37.1–42.4
Birthweight (g)	
Mean (SD)	3384 (401)
Range	2485–4400
Birthweight centile, n (%)	
0–3	0 (0)
3–10	4 (5)
10–25	14 (16)
25–50	20 (23)
50–75	27 (31)
75–90	16 (18)
90–97	6 (7)
97–100	0 (0)

*BMI*, body mass index; *GA*, gestational age; *SD*, standard deviation.

**Figure 4:**
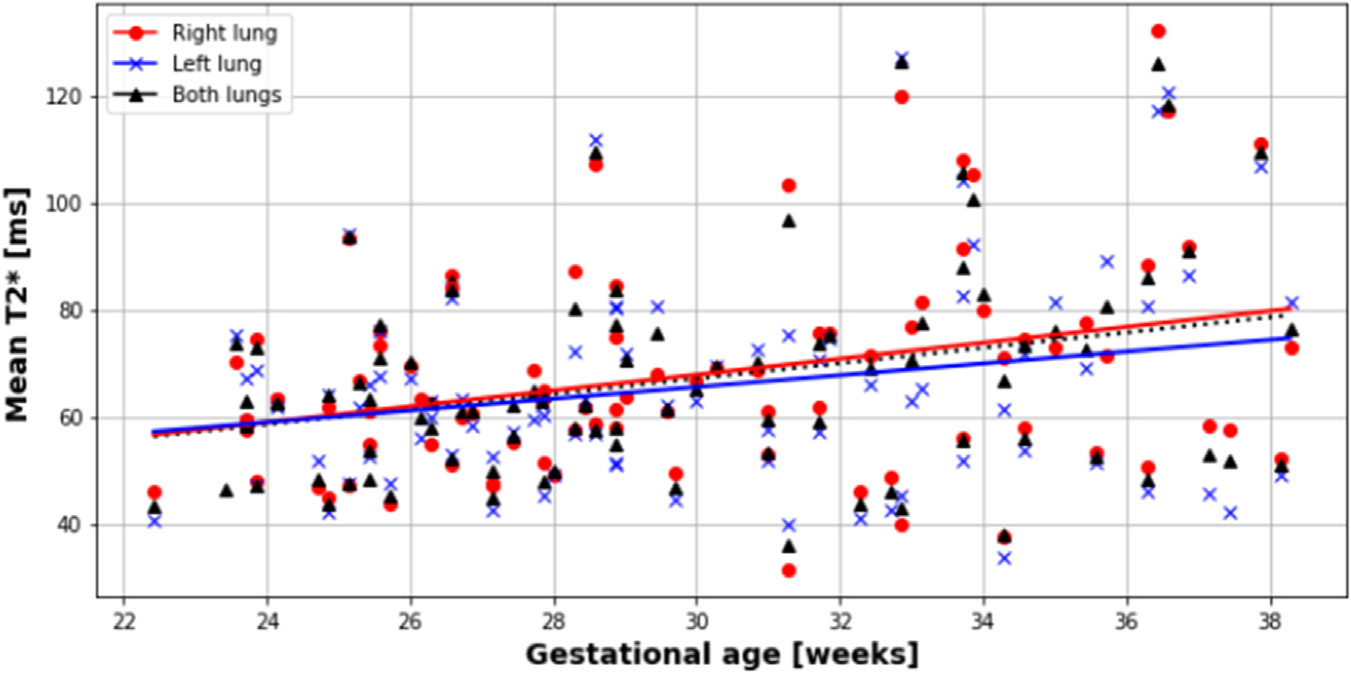
Mean pulmonary T2* values over GA at scan in right (*red*), left (*blue*) and both lungs (*black*)

**Figure 5:**
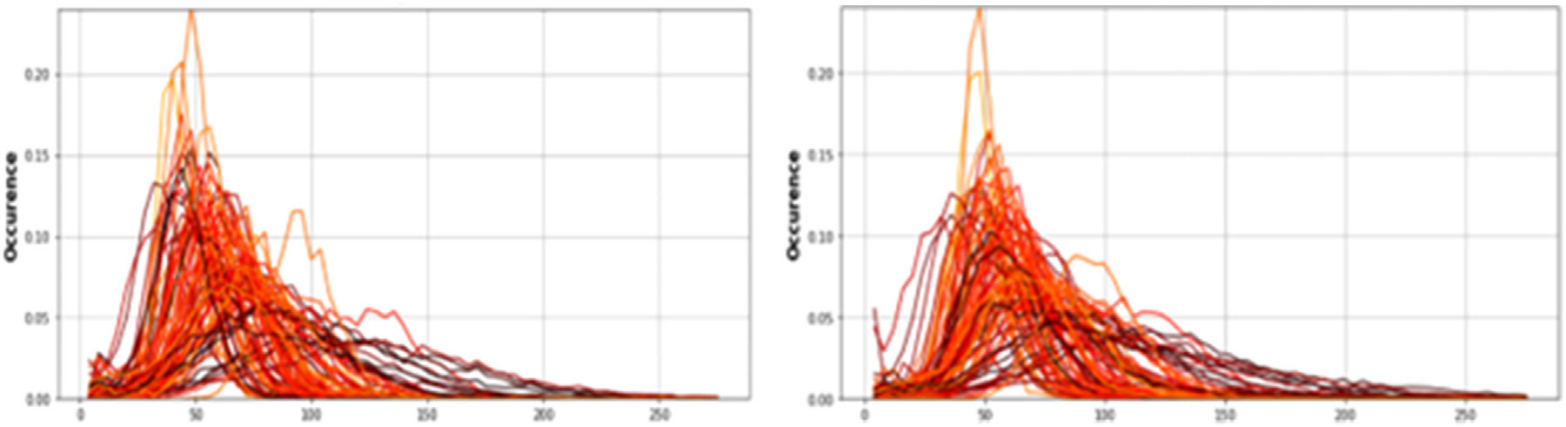
Histograms to illustrate the distribution of pulmonary T2* voxel values Figure illustrates the distribution of pulmonary T2* voxel values from the right (*right*) and left (*left*) lungs, from all participants, (*lighter curves* represent earlier gestational ages at scan)

**Figure 6:**
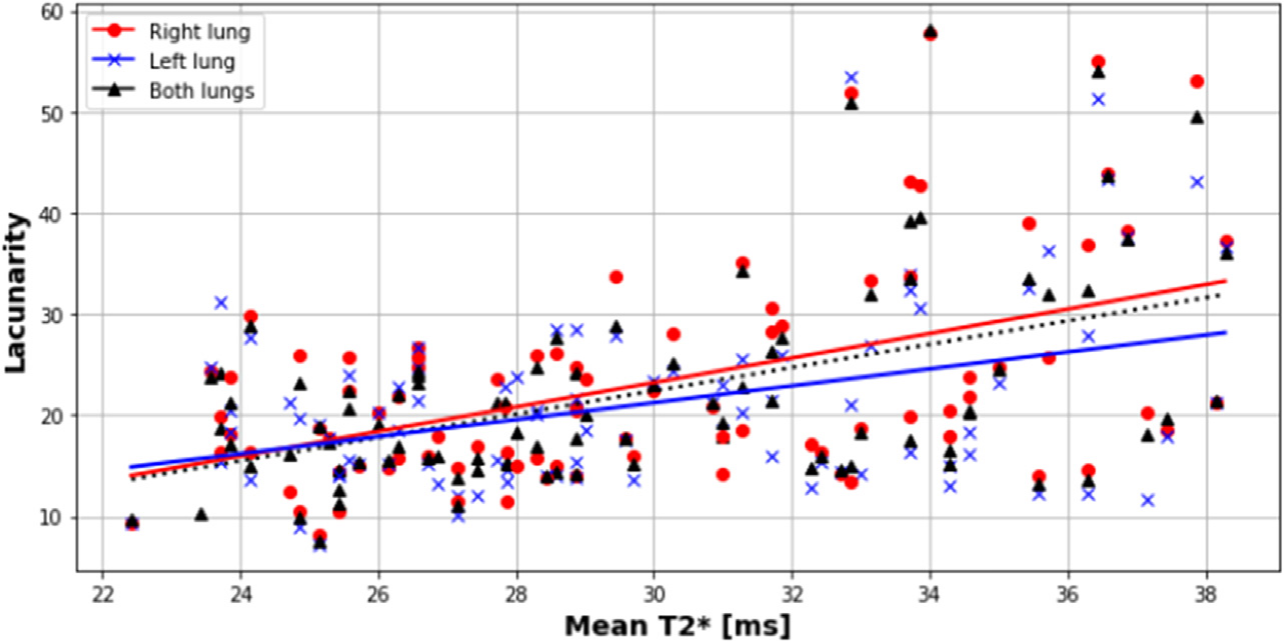
Scatterplot of lacunarity measure Scatterplot (derived from T2* mapping) for right (*red dot*), left (*blue cross*) and both lungs (black *triangle*)

**Figure 7:**
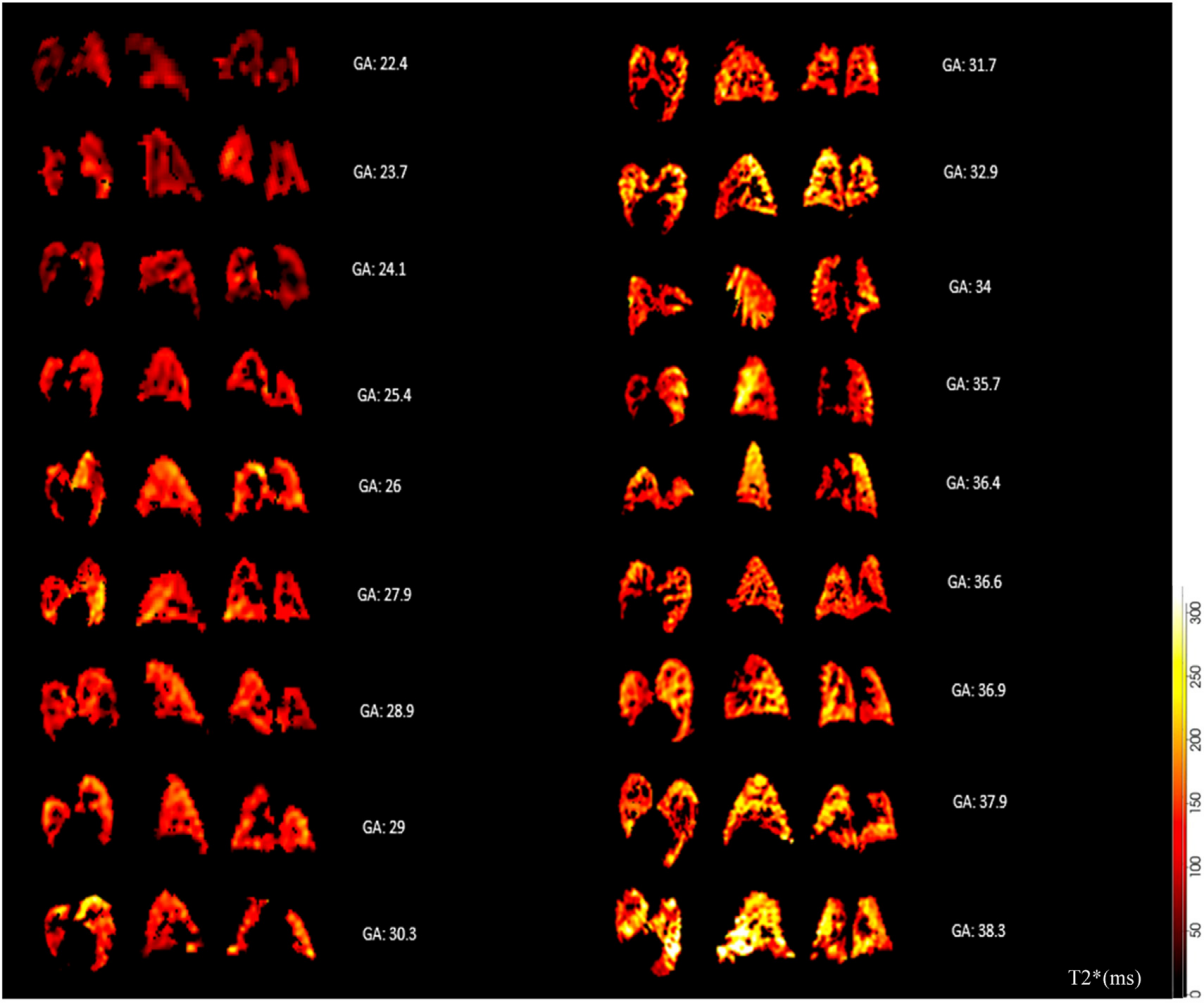
T2* maps in axial, sagittal and coronal views of fetal lungs across GA at scan (22.4-38.3 weeks) The scale of T2* values is displayed where the *red colour* is low and *yellow* is high.

**Figure 8:**
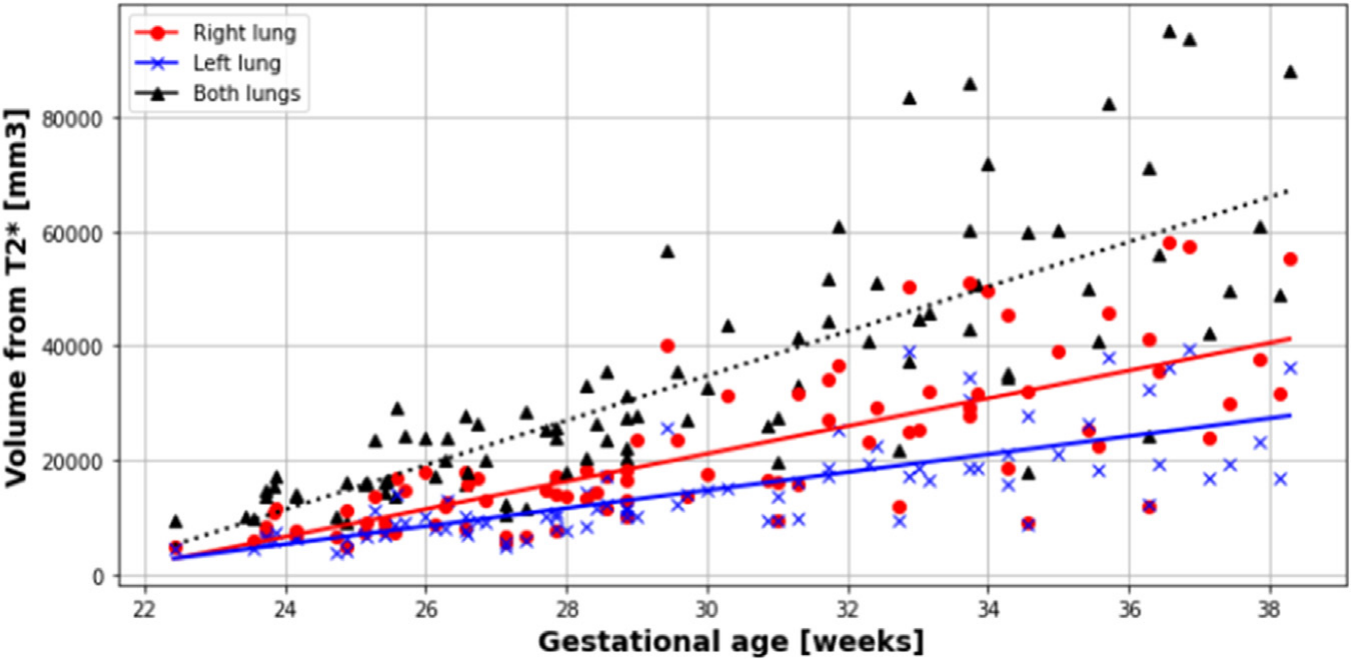
Pulmonary volumes over GA at scan in right (*red dot*), left (*blue cross*) and both lungs (*black triangle*)

Results for each lung, and both combined, were obtained from 20.6 to 38.3 weeks gestation. T2* values positively correlated with gestational age in each lung separately and combined, with no difference observed between the right and left lungs (*P*=.0001; slope=1.61; intercept=19.61; R^2^=.13), right lung (*P*=.005; slope=1.20; intercept=28.98; R^2^=.08) and left lung (*P*=.0006; slope=1.51; intercept=22.80; R^2^=.11) (Figure 4 and 5). Similarly, lacunarity scores increased with gestation (Figure 6) (both lungs (*P*<.001), right lung (*P*<.001) and left lung (*P*<.001).). Visual inspection of the maps reveals increasing heterogeneity with gestation (Figure 7). Finally, as expected, lung volumes increased with gestation (total, right and left lungs (*P*<.001, *P*<.001, and *P*<.001)) (Figure 8).

Avena-Zampieri. Assessment of normal pulmonary development. Am J Obstet Gynecol MFM 2023.

Avena-Zampieri. Assessment of normal pulmonary development. Am J Obstet Gynecol MFM 2023.

Avena-Zampieri. Assessment of normal pulmonary development. Am J Obstet Gynecol MFM 2023.

Avena-Zampieri. Assessment of normal pulmonary development. Am J Obstet Gynecol MFM 2023.

Avena-Zampieri. Assessment of normal pulmonary development. Am J Obstet Gynecol MFM 2023.

Intraobserver and interobserver variability was good for both volume and mean T2* values. Intraobserver variability had an intraclass correlation coefficient of 0.989 for volume and 0.998 for mean T2* and interobserver variability had an interclass correlation coefficient of 0.951 for volume and 0.997 for mean T2*.

## Discussion

### Significant findings and results

In 87 healthy pregnancies, we have demonstrated that antenatal mean pulmonary T2* values increase with increasing gestational age between 20 and 38 weeks gestation in right and left lungs separately, and both lungs combined. Pulmonary volumes were also found to increase with gestation with lower volumes in the left lung compared with the right (Figure 8).

These findings are in contrast with the only previously published study assessing pulmonary T2* values in the antenatal period.^13^ Sethi et al^13^ reported no variation in T2* values across gestation in total lung tissue. However, their sample size comprised only 9 fetuses, in comparison with the 87 included in our study; the gestational period assessed was also narrower (28–38 weeks, in comparison with 20.6–38.3 weeks); both factors may explain the differing findings. The mean T2* values appeared to be higher in our study for comparable gestational ages than in the work by Sethi et al.^13^ This may be explained in part, by the fact that our study was conducted on a 3T MRI system in contrast to a 1.5T used by Sethi et al.^13^ Higher field strength is associated with higher T2* values.^22^ Increased dephasing occurs at higher field strengths owing to susceptibility effects and increased inhomogeneity in the magnetic fields of the magnet. Differences in acquisition protocols, that is, TRs (repetition times) and TEs (time to echo), field of view and differing tools for image-based shimming, may also influence to a lesser extent the differences observed in T2* values between the 2 studies.

Unlike previous studies which utilized T2* to assess fetal lungs; and other structures, such as the brain,^11^ liver,^12^ spleen,^13^ and lungs,^13^ our study also accounted for fetal motion using the DSVR post-processing technique.^6^ Fetal MRI and quantitative T2* analysis are particularly susceptible to motion. Using raw datasets may mean that nonpulmonary tissue was included and smaller structures such as vessels may inadvertently have been part of the segmentations, thus rendering results inaccurate. Although the application of DSVR pipelines results in altered, higher tissue resolution which may affect the T2* values obtained, we found no statistical difference between mean T2* datasets obtained from the raw data and those obtained following the DSVR pipeline.

### Research and clinical implications

T2* values are obtained from the combined effects of spin-spin relaxation (T2) and magnetic field inhomogeneity,^8^ hence T2 and T2* values are inherently related. T2* values within tissues are attributable to the effect of magnetic field inhomogeneities, for example arising from the paramagnetic properties of deoxygenated hemoglobin in comparison with oxygenated hemoglobin. In addition, T2* values are also affected by elements of tissue composition (the intrinsic T2 value) such as cell density, water or fluid content, and surface area. An increase in T2* values in the fetal lungs with advancing gestational age may be partly related to altering tissue and fluid composition as gestation progresses. These findings are consistent with the increase of T2 values, attributed to developmental changes, in the fetal lungs from 19 to 40 weeks studied in 105 healthy pregnancies by Cannie et al.^23^

As gestation progresses, an increasing proportion of cardiac output is directed to the fetal lungs,^24^ ranging from 13% between 16 and 28 weeks to 30% between 28 and 36 weeks.^25^ Fetal hemoglobin concentration is constant from approximately 10 weeks until 24 weeks gestation. Although concentrations then decline toward term, adult hemoglobin concentrations increase from approximately 31 weeks.^26^ This might affect T2* values, similar to the changes in geometry related to the development of the pulmonary vascular tree, such as increased capillary density within each magnetic resonance (MR) voxel. Lacunarity scores reflect changes in granularity and tissue heterogeneity. We demonstrated a significant correlation with gestational age for both lungs, which again, may in part be related to the maturation of the pulmonary vascular tree reflected in the T2* values recorded.

A diverse range of processes are known to alter the MR properties of pulmonary parenchyma across gestation. Developmental processes include angiogenesis, development of the alveoli and bronchioles, and alterations of pulmonary tissue content. These require cellular proliferation and differentiation associated with an increased metabolic demand and tissue perfusion. In addition, the fluid composition of the lungs also alters with gestation, lung fluid secretion decreases, and sodium transport dependent resorption increases, which facilitates gaseous exchange after birth.^27^ An increase in surfactant production and therefore concentration of phospholipid, lipid, and protein content is also known to occur with increasing gestation. Maturation of the pulmonary structural components includes not only the microvasculature but also the extracellular matrix, composed of a complex mixture of fibrous proteins. Considering that the extracellular matrix governs many fundamental cellular processes (including alveolar septation), especially during the saccular phase, those processes might be reflected in the T2* values recorded. All these physiological changes may therefore also affect T2* values across gestation.

The pulmonary volumes obtained in our study were lower than previously reported;^28^, ^29^, ^30^ however, this is most likely attributable to the conservative segmentation deployed to ensure only pulmonary tissue was included. We therefore propose that where absolute lung volumes are required, these are optimally obtained from T2 datasets as opposed to the T2* datasets.

### Strengths and limitations

This study characterizes pulmonary development across gestation using mean T2* encompassing 87 healthy pregnancies between 20.6 and 38.3 weeks gestation. Utilizing DSVR post-processing pipelines facilitated correction for motion and distortion deterioration. We have demonstrated excellent reliability scores, making this technique reproducible and valuable for future applications. However, assessment of pulmonary T2* values, provides an indirect assessment of tissue perfusion only and the application of DSVR may also alter the mean values of T2* owing to interpolation effects applied during the super resolution as it attempts to reconstruct the original scene image with high resolution. Although a large gestational age range has been encompassed within this study, no datasets were included before 20 weeks or after 39 weeks gestation. Further work focusing on these periods should be conducted to provide a complete assessment of in utero pulmonary development. It should also be noted that most women who participated in this study are White. It is therefore imperative that further work should assess a diverse population to ensure that findings are applicable to all ethnic groups.

### Conclusion and clinical implications

Our results have given functional insight into pulmonary development potentially reflecting increased perfusion and metabolic activity of tissue as gestation advances. Investigation of fetuses at high risk of pulmonary morbidity using these techniques will be required to assess if alterations in pulmonary T2* values are associated with pathology. If this is found to be the case, the role of T2* as a prognostic marker can then be assessed.

## Appendix Supplementary materials

Image, application 1

Image, application 2
