# Supplementary material for: Assessment of normal pulmonary development using functional magnetic resonance imaging techniques
Source: Am J Obstet Gynecol MFM. 2023 Jun;5(6):100935. doi: 10.1016/j.ajogmf.2023.100935 (PMC10711505; doi:10.1016/j.ajogmf.2023.100935)
Supplement: Supplementary file 1 [file mmc1.docx]

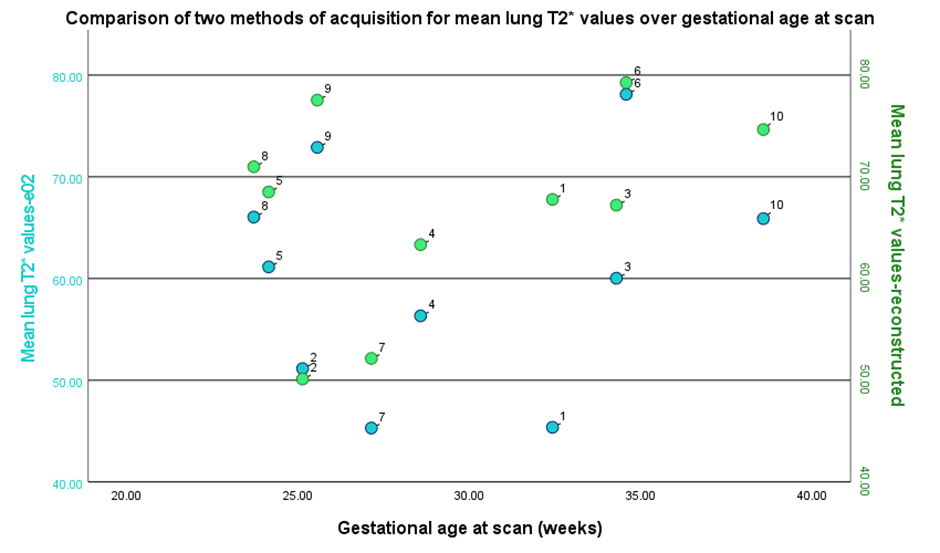


Supplementary figure 1: Comparison of mean pulmonary T2* values obtained after 3D reconstruction (green) and raw stacks (blue) for each of the 10 cases.
