# Supplementary material for: Assessment of normal pulmonary development using functional magnetic resonance imaging techniques
Source: Am J Obstet Gynecol MFM. 2023 Jun;5(6):100935. doi: 10.1016/j.ajogmf.2023.100935 (PMC10711505; doi:10.1016/j.ajogmf.2023.100935)
Supplement: Supplementary file 2 [file mmc2.docx]

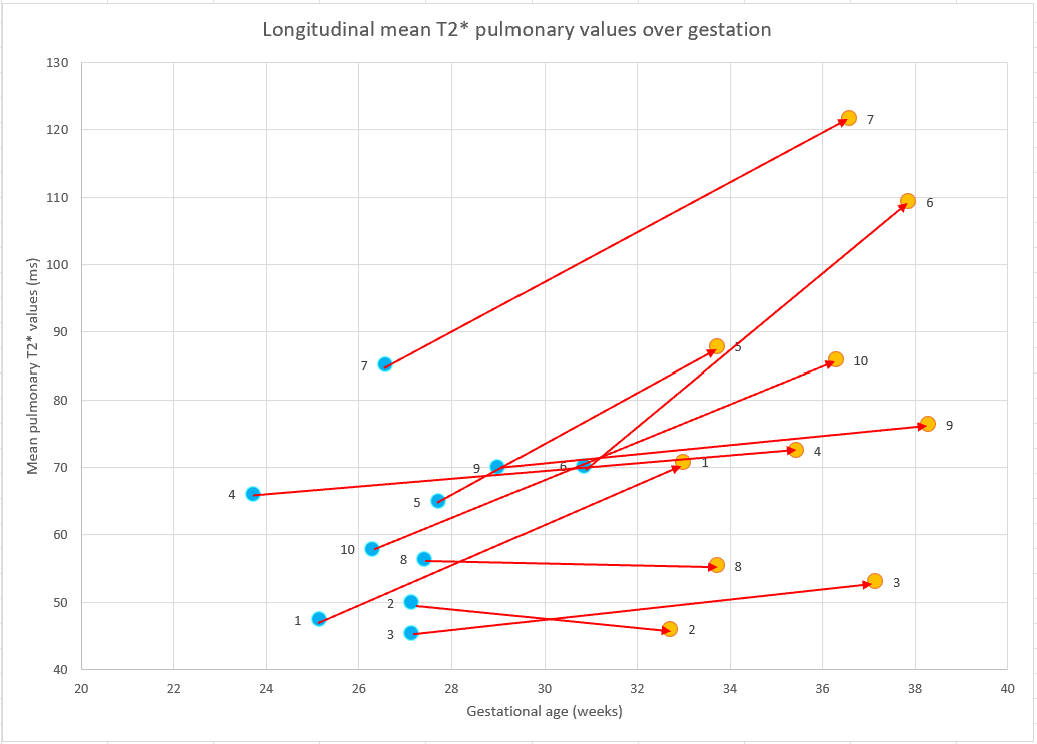


Supplementary figure 2: Longitudinal data of 10 of the cases over gestation with a repeated scan in the course of their pregnancy. The blue dots represent the first scans and the green the second scans.
